# A Case of a Chronic Ectopic Pregnancy in a Patient With Negative Serum β‐HCG

**DOI:** 10.1002/ccr3.72992

**Published:** 2026-06-23

**Authors:** Shushu Yu, Xiaohan Su, Lanxin Liu, Xianjing Wang, Xinliang Chen

**Affiliations:** ^1^ Department of Obstetrics and Gynecology International Peace Maternity and Child Health Hospital, School of Medicine, Shanghai Jiao Tong University Shanghai China; ^2^ Shanghai Key Laboratory of Embryo Original Disease Shanghai China

**Keywords:** chronic ectopic pregnancy, clinical diagnosis, histopathological diagnosis, negative serum β‐human chorionic gonadotropin

## Abstract

Chronic ectopic pregnancy should be considered in women with adnexal masses and persistent abdominal pain, even when serum β‐hCG is negative, to prevent delayed diagnosis. This article reports a case of ectopic pregnancy with negative serum β‐hCG levels nearly 3 months after curettage.

**Abbreviations**
CDFI: color Doppler flow imaging
CEP: chronic ectopic pregnancy
MRI: magnetic resonance imaging
TVUS: transvaginal ultrasonography
β‐HCG: β‐human chorionic gonadotropin

## 1 Introduction

Ectopic pregnancy is defined as the implantation and development of a conceptus outside the uterine cavity [1]. Approximately 1%–2% of pregnancies are considered as ectopic, though the true prevalence remains uncertain and may be higher [2, 3]. Ectopic pregnancy is the leading cause of death in early pregnancy, accounting for approximately 4% of all pregnancy‐related deaths [4]. In chronic ectopic pregnancies (CEP), serum β‐human chorionic gonadotropin (β‐HCG) levels are frequently low or even negative. It accounts for approximately 6%–20% of ectopic pregnancy cases. Chronic adhesions develop around the lesion due to recurrent inflammation [5]. CEP is a relatively rare type of ectopic pregnancy that typically develops due to delayed diagnosis and treatment of ectopic pregnancy [6]. The diagnosis of CEP is challenging due to its atypical clinical manifestations and laboratory findings, often leading to misdiagnosis or a missed diagnosis. Here, we present a case of CEP with negative serum and urine β‐hCG levels, which was preoperatively diagnosed as an adnexal tumor. This report aims to improve the clinical awareness of this condition. We present the following case in accordance with the CARE guideline.

## 2 Case Presentation

### 2.1 Patient Profile

The patient was a 33‐year‐old, Han Chinese woman with a reproductive history of gravida 4, para 1 (G4P1), including three induced abortions and one cesarean section. She reported regular 28‐day menstrual cycles with moderate flow and no dysmenorrhea. In September 2025, she presented to our hospital because of an unintended pregnancy, and serum β‐hCG testing was performed to confirm her pregnancy status. On September 28, 2025, the serum β‐hCG level was 22,555 IU/L. She subsequently underwent ultrasonography at an outside hospital, which reportedly did not demonstrate definite sonographic features of ectopic pregnancy, and uterine curettage was performed there on September 30, 2025. Postoperative histopathological examination revealed decidual tissue and scant highly secretory endometrium, with no significant villous tissue identified. A follow‐up serum β‐hCG test on October 9, 2025, showed a level of 7237 IU/L. However, further serial β‐hCG monitoring was not completed because of the patient's work commitments. Following the procedure, she reported no sexual intercourse. She later stated that menstruation resumed on November 27, 2025, with normal flow and color. Recurrent dull pain in the left lower abdomen began on the same date but was not initially considered significant by the patient. On December 4, 2025, she presented to another hospital for further evaluation. Serum β‐hCG was negative. Transvaginal ultrasonography (TVUS) revealed a 4 cm pelvic mass. She was subsequently referred to and presented at our hospital on December 5, 2025. Pelvic contrast‐enhanced magnetic resonance imaging (MRI) performed on the same day showed a hemorrhagic lesion arising from the left adnexal region, suggestive of a benign or borderline tumor. A follow‐up TVUS at our hospital on December 9, 2025, demonstrated a mixed echogenic area adjacent to the left ovary, measuring approximately 3.7 × 4.1 × 4.3 cm with ill‐defined borders. Color Doppler flow imaging (CDFI) showed no significant internal vascularity. Serum β‐hCG retested at our hospital on December 9, 2025, was negative. All tumor markers assessed on December 10, 2025, were within normal limits. She was admitted for diagnostic laparoscopy based on the clinical presentation of recurrent left lower abdominal pain and imaging findings suggestive of a benign or borderline left adnexal mass.

### 2.2 Gynecological Examination and Laboratory Tests

#### 2.2.1 Gynecological Examination Revealed the Following Findings

- Vulva: Nulliparous appearance.
- Vagina: Patent. Cervix: Smooth, with no cervical motion tenderness.
- Uterus: Anterior, moderately enlarged and non‐tender.
- Adnexa: A 4‐cm, mildly tender, and mobile mass was palpated in the left adnexal region. The right adnexal region was unremarkable.

#### 2.2.2 Relevant Laboratory Test Results Were as Follows

- December 5, 2025 (external hospital): Serum β‐hCG, negative.
- December 9, 2025 (our hospital): Serum β‐hCG, negative.
- December 10, 2025 (our hospital): Tumor markers, all within normal limits.
- December 20, 2025 (our hospital): Urine β‐hCG, negative.

### 2.3 Ultrasound Examination

TVUS performed on December 9, 2025, revealed a mixed‐echogenicity area adjacent to the left ovary, measuring approximately 3.7 × 4.1 × 4.3 cm with ill‐defined borders. CDFI demonstrated no significant internal vascularity.

### 2.4 Pelvic MRI Examination

**FIGURE 1:**
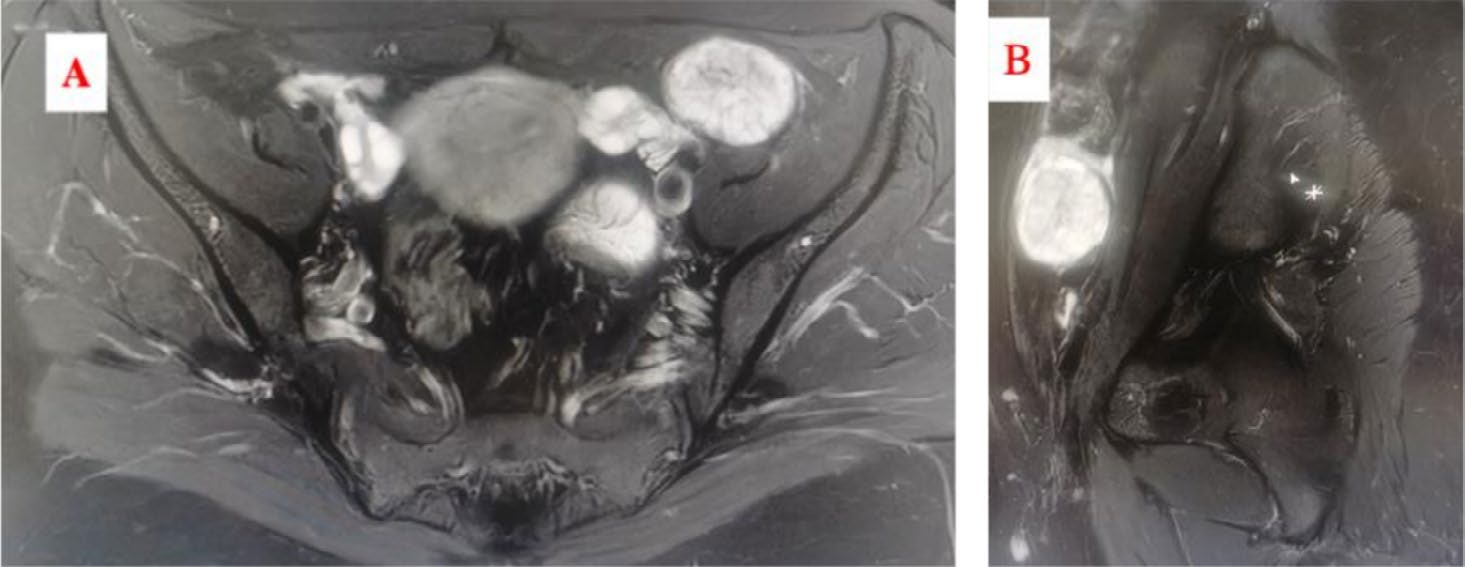
MRI demonstrated an inhomogeneous ectopic mass in both the (A) axial and (B) coronal section.

An MRI conducted on December 5 revealed a round lesion in the left adnexal region. On T1‐weighted images, the lesion exhibited heterogeneous signal intensity, including hyperintense, isointense, and hypointense components. On T2‐weighted images, the signal pattern was also heterogeneous, consisting of hyperintense, mildly hyperintense, and hypointense areas. Multiple cystic components were noted within the lesion, which measured approximately 3.6 × 2.7 cm. After intravenous contrast administration, the lesion showed heterogeneous enhancement. The ipsilateral left ovary was identified separately from the lesion. In summary, the imaging findings were consistent with a hemorrhagic lesion in the left adnexal region of indeterminate origin, suggesting a benign or borderline entity (Figure 1).

### 2.5 Surgical Findings and Pathological Diagnosis

**FIGURE 2:**
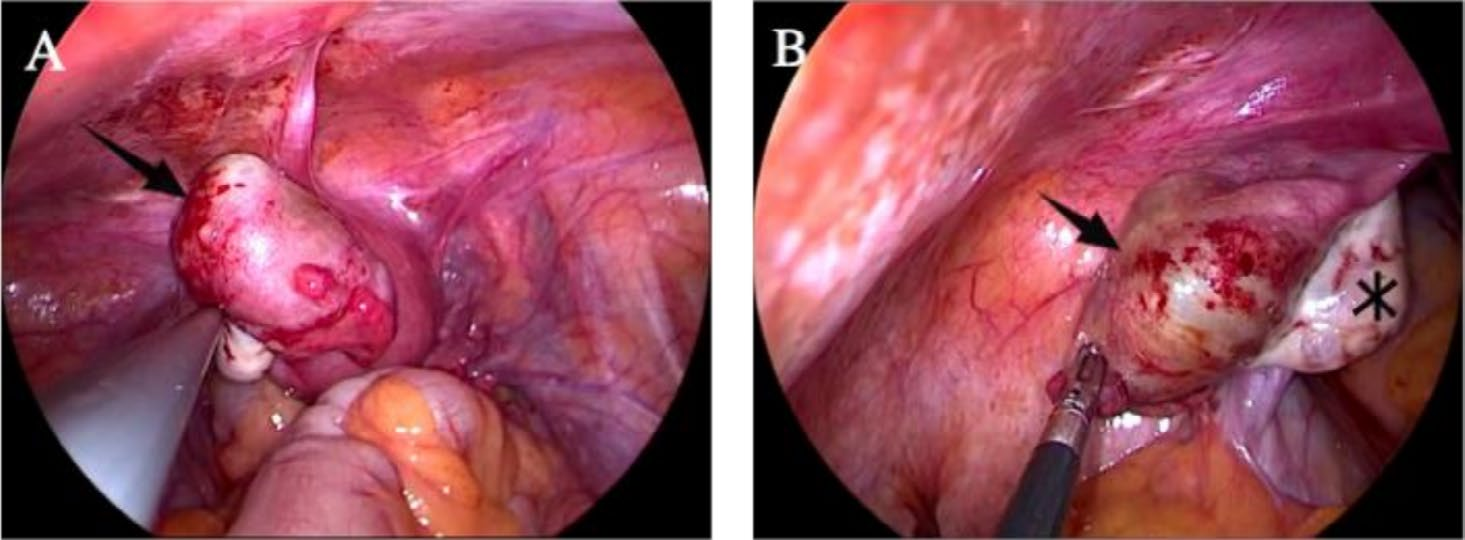
Laparoscopic view showing an exophytic mass (arrow) on the left fallopian tube. The left ovary (asterisk) looks normal.

Intraoperatively, the uterus appeared normal in size and morphology. The left fallopian tube was significantly dilated and thickened at the ampullary and fimbrial segments, forming a mass approximately 4 × 3 × 3 cm in size. Its surface was purplish‐blue, and there was no evidence of rupture. The fimbriated end of the left fallopian tube was sealed (Figure 2).

Postoperative histopathological examination of the resected specimen confirmed the diagnosis of chronic ectopic pregnancy. The left fallopian tube lumen was markedly dilated and contained an extensive organized hematoma. Within the hematoma, highly degenerated chorionic villi were identified. The tubal wall exhibited significant chronic inflammatory cell infiltration and fibrosis.

## 3 Discussion

In a normal intrauterine pregnancy, trophoblastic cells secrete β‐hCG, with serum levels typically reaching 50–300 IU/L within 2 weeks post‐conception. Indeed, β‐hCG becomes detectable in both urine and serum as early as 16 days after the luteinizing hormone surge [7]. Qualitative urinary pregnancy tests offer a rapid and simple method for detecting β‐hCG, demonstrating approximately 99% sensitivity when β‐hCG levels exceed 25 IU/L [8]. In most cases of ectopic pregnancy, the combination of amenorrhea, vaginal bleeding, abdominal pain, a pelvic or adnexal mass, and positive β‐hCG findings facilitates diagnosis.

However, chronic ectopic pregnancy (CEP) differs substantially from the more typical presentation of ectopic pregnancy. The classic presentation of CEP includes prolonged irregular vaginal bleeding, mild lower abdominal pain after a period of amenorrhea, a palpable pelvic mass, slightly elevated serum β‐hCG levels, and decidual change in the endometrium. By contrast, atypical CEP may present without severe abdominal pain, without obvious amenorrhea, with negative serum β‐hCG, and with endometrial histology that resembles a normal menstrual cycle. This diagnostic challenge is particularly pronounced in patients with indolent bleeding and only mild abdominal pain.

A large series analyzed by Clemens et al., including 399 cases of CEP, showed that 32% of patients had negative β‐hCG, 71% reported abdominal pain, 18% were asymptomatic, and 48% had adnexal masses on ultrasound [6]. Accordingly, CEP is frequently misdiagnosed preoperatively as chronic pelvic inflammatory disease, ovarian neoplasm, or a broad ligament tumor. Demir et al. reported another case of CEP in a 34‐year‐old woman whose serum β‐hCG level declined from 4000 to 7.3 mIU/mL; MRI demonstrated a hematoma on axial T1‐weighted images, with a low‐signal yolk sac surrounded by high‐signal subacute hemorrhage [9]. On contrast‐enhanced pelvic MRI, characteristic findings of CEP may include a tubular adnexal lesion with wall enhancement, hemorrhagic signal along its course, and internal patchy areas of high signal intensity. Misdiagnosis may also occur at rare implantation sites. Abraham et al. described a β‐hCG‐negative old ovarian pregnancy in which laparotomy revealed an 8 × 6 cm ovarian mass containing a macerated fetus [10]. Similarly, Sherin et al. reported a woman in her 30's with progressive lower abdominal pain, inconclusive pregnancy tests, and spontaneous vaginal bleeding who was ultimately diagnosed with CEP on histopathological examination [5].

In clinically equivocal cases, Hung et al. emphasized that diagnostic laparoscopy and histopathological examination remain crucial [11]. Because functional trophoblastic tissue is often absent or markedly degenerated in CEP, serum β‐hCG levels are frequently very low or undetectable. Laparoscopy is generally considered the preferred initial diagnostic and therapeutic approach, as conservative surgery is often precluded by severe tubal damage [11]. In addition, CEP is usually less responsive to methotrexate, likely because of minimal residual active trophoblastic tissue or the absence of viable chorionic villi [6]. Nevertheless, laparoscopic management can be technically challenging because dense pelvic adhesions often surround the implantation site. Adhesiolysis therefore carries risks of bowel or bladder injury and ureteral damage. When the ectopic lesion is densely adherent to the ipsilateral ovary, meticulous dissection is essential to preserve ovarian tissue and avoid inadvertent oophorectomy [12].

The present case highlights several important diagnostic pitfalls. The patient developed recurrent left lower abdominal pain after curettage but initially interpreted this as the resumption of menstruation. More importantly, repeated serum and urine β‐hCG tests were negative, which reduced clinical suspicion for a pregnancy‐related disorder. An adnexal mass was identified more than 2 months after the D&C, and pelvic MRI favored a benign or borderline lesion, further shifting the preoperative diagnostic impression away from ectopic pregnancy.

An additional diagnostic issue in this case is the localization of the antecedent pregnancy. In September 2025, serum β‐hCG was measured because the patient presented with an unintended pregnancy. According to the available history, ultrasonography performed at the outside hospital before curettage did not demonstrate definite sonographic features of ectopic pregnancy. However, the absence of sonographic evidence of ectopic pregnancy did not definitively establish an intrauterine pregnancy, particularly because the curettage specimen subsequently showed decidual tissue and scant highly secretory endometrium without significant chorionic villi. Therefore, the earlier pregnancy event may retrospectively be regarded as a pregnancy of unconfirmed location, or possibly a pregnancy of unknown location. This distinction is clinically important, because the initial D&C did not conclusively exclude ectopic pregnancy.

It is also important to distinguish CEP from persistent ectopic pregnancy. Persistent ectopic pregnancy generally refers to continued viable ectopic trophoblastic tissue after treatment for a known ectopic pregnancy and is usually associated with persistently abnormal β‐hCG levels. In contrast, CEP is characterized by repeated minor bleeding, degeneration of chorionic villi, chronic inflammatory reaction, and often very low or even negative β‐hCG levels. In the present case, no ectopic pregnancy had been diagnosed or treated before surgery, and the subsequent course was dominated by chronic pelvic pain, a persistent adnexal/pelvic mass, and repeatedly negative β‐hCG results. For these reasons, CEP is considered the more appropriate final diagnosis, although the antecedent pregnancy was not definitively localized.

The preoperative misdiagnosis in this case can therefore be attributed to several interacting factors rather than to a single cause. First, repeated negative pregnancy tests led clinicians to prematurely downplay the possibility of a pregnancy‐related disorder. Importantly, in CEP, a negative β‐hCG result does not necessarily represent a testing error; rather, it may reflect advanced degeneration of trophoblastic tissue to a level below the assay detection threshold. Second, the pathology report from the initial curettage was not fully integrated into the early diagnostic reasoning. Retrospective review showed that no definite chorionic villi had been identified, which should have raised concern for ectopic pregnancy or pregnancy of unconfirmed location. Third, the imaging findings were interpreted with disproportionate weight, and the benign‐appearing MRI impression likely contributed to anchoring bias, diverting attention toward adnexal neoplasm or inflammatory mass rather than occult ectopic pregnancy. Thus, this case illustrates how atypical presentation, incomplete pregnancy localization, and cognitive bias may together delay the diagnosis of CEP.

This case also provides several practical clinical lessons. First, when curettage is performed for presumed intrauterine pregnancy loss, clinicians should carefully assess whether chorionic villi are definitively identified in the curettage specimen, and the pathology report should be reviewed closely for the presence or absence of villi. If no definite chorionic villi are identified, ectopic pregnancy or pregnancy of unconfirmed location should be strongly considered. Second, serum β‐hCG should be monitored serially after curettage until it becomes negative, and a slow decline or plateau should prompt further evaluation for retained products of conception or ectopic pregnancy, including CEP. Third, serial β‐hCG monitoring alone may still be insufficient to exclude CEP, because degenerated chorionic villi may produce very low or undetectable β‐hCG levels. Therefore, a safer and more reliable follow‐up strategy after curettage may be the combination of serial serum β‐hCG measurement and gynecological ultrasonography. Ultrasound can directly evaluate the endometrium for retained products of conception and the adnexa for any persistent or evolving mass, thereby improving the likelihood of detecting occult ectopic pregnancy and reducing delayed diagnosis and unnecessary morbidity.

## 4 Conclusion

This case further emphasizes the essential significance of acquiring a comprehensive medical history, performing a meticulous physical examination, and maintaining a high index of clinical suspicion to avoid diagnostic errors. It also highlights the diagnostically challenging nature of chronic ectopic pregnancy, which may present with non‐specific symptoms, inconclusive physical findings, and equivocal or even negative β‐hCG results. Therefore, in women of reproductive age with a recent history of pregnancy‐related events and a suspected pelvic mass, a detailed preoperative history and a broad differential diagnosis are essential. Importantly, in women with a recent history of pregnancy of unconfirmed location who later present with pelvic pain, a persistent adnexal or pelvic mass, or unexplained symptoms, chronic ectopic pregnancy should be considered even when serum β‐hCG is negative. After curettage, careful pathological review for chorionic villi, serial β‐hCG follow‐up to negativity, and gynecological ultrasonography should be considered to exclude retained products of conception and occult ectopic pregnancy, thereby reducing delayed diagnosis and unnecessary morbidity.

## Author Contributions

**Xiaohan Su:** methodology, software, investigation. **Shushu Yu:** conceptualization, data curation, writing – original draft. **Xinliang Chen:** conceptualization, supervision, project administration, visualization, resources, writing – review and editing. **Xianjing Wang:** conceptualization, funding acquisition, supervision, project administration, resources, writing – review and editing. **Lanxin Liu:** validation, formal analysis.

## Funding

The authors have nothing to report.

## Consent

Written informed consent was obtained from the patient for publication of this case report and any accompanying images. A copy of the written consent is available for review by the Editor‐in‐Chief of this journal.

## Conflicts of Interest

The authors declare no conflicts of interest.

## Data Availability

The data that support the findings of this study are available on request from the corresponding author. The data are not publicly available due to privacy or ethical restrictions.
